# Supplementary material for: Long noncoding RNA hottip maintained skeletal homeostasis via suppressing the enhancer of zeste homolog 2 (Ezh2)/histone methylation regulatory axis
Source: Noncoding RNA Res. 2025 Feb 28;12:141–51. doi: 10.1016/j.ncrna.2025.01.003 (PMC11985131; doi:10.1016/j.ncrna.2025.01.003)
Supplement: Multimedia component 1 [file mmc1.pdf]

Table1 Primer sequences for qPCR	
Gene	Sequence
ALP	5’- GCCCTCTCCAAGACATATA -3’<br>5’- CCATGATCACGTCGATATCC-3’
OSX	5’- GTGAATTCACCTTTCAGCCCCAAAACC-3’<br>5’- TGGGATCCCAGCTGTGAATGGGCTTCTT-3’
OCN	5’- CCTCAGTCCCCAGCCCAGATCC-3’<br>5’-CAGGGCAGAGAGAGAGGACAGG-3’
OPN	5’-TCACCATTCGGATGAGTCTG-3’<br>5’-ACTTGTGGCTCTGATGTTCC-3’
RUNX2	5’-GACTGTGGTTACCGTCATGGC-3’<br>5’-ACTTGGTTTTTCATAACAGCGGA-3’
BMP2	5’-AGTTCTGTCCCCAGTGACGAGTTT-3’<br>5’-GTACAACATGGAGATTGCGCTGAG-3’
Arg-1	5’-ATCCCAGCAGTTCCTTTCTG-3’<br>5’-CATCTTTTGAACAGCGTGGA-3’
Atp6V0D2	5’-CTGGTTCGAGGATGCAAAGC-3’<br>5’-GTTGCCATAGTCCGTGGTCTG-3’
CTSK	5’-CTCGGCGTTTAATTTGGGAGA-3’<br>5’-TCGAGAGGGAGGTATTCTGAGT-3’
TRAP	5’-TCATGGGTGGTGCTGCT-3’<br>5’-GCCCACAGCCACAAATCT-3’
NF-κB	5’-GGAGAGTCCGAGAATCGAGAT-3’<br>5’-TTGCAGCTAGGAAGTACGTCT-3’
RANKL	5’-CCAGGAGAGGCATTATGAGCA-3’<br>5’-ACTGTCGGAGGTAGGAGTGC-3’
β3-intergrin	5’-CAGTGGCCGGGACAACTC-3’<br>5’-GACAAAGTCTCATCTGAGCACCAG-3’
Dc-stamp	5’-AAGGTGGTGGCGTTATACTGC-3’<br>5’-CTGGCACAGCGGATGTGAG-3’
BCL-6	5’-GACGTTGTCATCGTGGTGAG-3’<br>5’-GGTTGCATTTCAACTGGTCA-3’
Hottip	5’-AAAGGTGTAGCAAGCCCCTC-3’<br>5’-TGATATAACCCTCCCCCGCA-3’

Table2 Primer sequences for CHIP-PCR	
Gene	Sequence
RUNX2	5'-TGCCATGCGCTCCTGAATTA-3'<br>5'-GGAAGGTGGCCAAGAAGGAA-3'
ALP	5'-TTCCTGACCCAGAGACACCA-3'<br>5'-CCCCAAGGAGTCACTGAACC-3'
OSX	5'-CTTCCCTGCGGTCGGTTTAT-3'<br>5'-TCGGGGTCTCATGTCAGTCT-3'
Balap	5'-CACGGGATACACACACCCTC-3'<br>5'-AAGGAGTTGTGCTGGAGTGG-3'
